# From Envelope Spectra to Bearing Remaining Useful Life: An Intelligent Vibration-Based Prediction Model with Quantified Uncertainty

**DOI:** 10.3390/s24227257

**Published:** 2024-11-13

**Authors:** Haobin Wen, Long Zhang, Jyoti K. Sinha

**Affiliations:** 1 Dynamics Laboratory, The Department of Mechanical and Aerospace Engineering, The University of Manchester, Manchester M13 9PL, UK; haobin.wen@manchester.ac.uk; 2 The Department of Electrical and Electronic Engineering, The University of Manchester, Manchester M13 9PL, UK; long.zhang@manchester.ac.uk

**Keywords:** bearings, remaining useful life, vibration, prognostics and health management, variational autoencoder, uncertainty quantification, physical interpretation

## Abstract

Bearings are pivotal components of rotating machines where any defects could propagate and trigger systematic failures. Once faults are detected, accurately predicting remaining useful life (RUL) is essential for optimizing predictive maintenance. Although data-driven methods demonstrate promising performance in direct RUL prediction, their robustness and practicability need further improvement regarding physical interpretation and uncertainty quantification. This work leverages variational neural networks to model bearing degradation behind envelope spectra. A convolutional variational autoencoder for regression (CVAER) is developed to probabilistically predict RUL distributions with confidence measures. Enhanced average envelope spectra (AES) are used as network input for its physical robustness in bearing condition assessment and fault detection. The use of the envelope spectrum ensures that it contains only bearing-related information by removing other rotor-related frequencies, hence it improves the RUL prediction. Unlike traditional variational autoencoders, the probabilistic regressor and latent generator are formulated to quantify uncertainty in RUL estimates and learn meaningful latent representations conditioned on specific RUL. Experimental validations are conducted on vibration data collected using multiple accelerometers whose natural frequencies cover bearing resonance ranges to ensure fault detection reliability. Beyond conventional bearing diagnosis, envelope spectra are extended for statistical RUL prediction integrating physical knowledge of actual defect conditions. Comparative and ablation studies are conducted against benchmark models to demonstrate their effectiveness.

## 1. Introduction

The remaining useful life (RUL) of a system is an important reliability metric representing the duration from the current time to the occurrence of failure when replacement is then needed. As a random variable conditioned on the present state of the system, it is distinguished from conventional metrics such as mean time to failure [1,2]. The prediction of RUL, often referred to as fault prognosis, is one of the primary tasks in practical prognostics and health management (PHM) for assessing future conditions of existing defects, which closely follows fault detection (detecting the onset of a fault) and fault diagnosis (identifying the mode, location, and root cause of a fault) [3,4]. In modern industrial systems, rolling element bearings (REBs) serve critical roles in a wide range of rotating machinery for motion transmission, friction reduction, and load support between moving parts. Given their critical functions, any faults within REBs could have a considerable impact on the health condition of the system as they propagate to other structural components. Hence, while many PHM techniques have targeted the early detection of bearing faults, the prediction of bearing RUL is also significant for preventing unscheduled overhauls, reducing downtime losses, and mitigating safety hazards through predictive maintenance.

To date, research efforts made towards bearing RUL prediction can be categorized into physics-based methods, data-driven methods, and their integration. On the one hand, physics-based methods involve precise modelling of bearing degradation dynamics [5], such as the association of contact stress with bearing fatigue life [6]. RUL prediction is realized by extrapolating the residual time or operation cycles for the physical variable to exceed a failure threshold, which could be highly accurate and interpretable if the model precisely reflects degradation processes. In other words, their performance relies on a prior and profound understanding of bearing systems and failure dynamics, which are admittedly difficult to access [7].

On the other hand, data-driven methods provide viable alternatives for learning the implicit relationship between condition monitoring (CM) data and RUL. One conventional strategy within this category involves formulating bearing RUL prediction as a degradation state estimation problem, wherein statistical health indicators (HI), e.g., vibration root-mean-square (RMS) values, can be extracted from measurements and recursively used for tracking the underlying dynamics via Bayesian estimators, such as Extended Kalman filter (EKF) or particle filter. Theoretically, Bayesian filters can be used to estimate the posterior probability density (PDF) of degradation states and extrapolate the end-of-life with uncertainty measures. However, they depend heavily on the accuracy of state transition models and measurement models to provide effective a priori estimates and handle measurement noise. In [2], the envelope spectral indicator was developed with a Bayesian filter to mitigate the effect from rotor-dynamics and more closely reveal actual defect conditions, which meanwhile improves the physical interpretability of the HI. Still, given time-variant bearing degradation dynamics, state estimation may be inadequate to capture complex and nonlinear behaviours of His, especially with a single stochastic model. To address the challenge, emerging techniques have resorted to machine learning (ML) by optimizing the performance measure of an RUL regression task based on available life-cycle data, providing a flexible approach for modelling bearing degradation. Among them, deep learning (DL) models have gained significant attention for their excellence in fitting nonlinear and implicit relationships.

In a typical modular DL model, RUL prediction tasks are broken down into two stages, i.e., the HI construction stage and the RUL estimation stage. Peng et al. introduced a reinforcement learning approach for HI construction using multisensory measurements, followed by a degradation state estimation module using the general mix-effect model [8]. Alternatively, two individual networks can be separately trained for these two stages [9]. Despite the benefit of specialization at each stage, one should note the propagation and amplification of prediction errors due to the sequential dependency of different modules. On the contrary, end-to-end DL models learn to provide direct RUL predictions from data with potentially improved overall performance. The multi-layer perceptron (MLP) network, or artificial neural network (ANN), has been applied by Felix Heimes [10] as a function estimator for directly predicting remaining operation cycles based on multiple input parameters, where an EKF-based solver was employed for updating network parameters. Noting the noise in measurement data, the same author further introduced the recurrent neural network for learning temporal mappings spanning multiple time steps in RUL prediction [10]. Given the limitation of MLP in capturing spatial dependencies and computation efficiency, the convolution neural network (CNN), along with the rectifier linear units and the pooling operation, was first introduced by Li et al. to PHM for RUL prediction using multi-dimensional input parameters [11]. To investigate the correlation between multi-sensor measurements, a deep separable convolution neural network is adopted to predict bearing RUL based on multi-channel vibration signals [12]. To enhance model fitting capability, sophisticated network structures and advanced training strategies have been introduced to bearing prognosis, such as graph neural network [13,14], transfer learning and domain adaptation [15], and federated learning [16], where outstanding performance is demonstrated through renowned bearing life-cycle datasets [17,18]. However, the common trait of these models is that they only provide deterministic or point prediction of RUL, whereas the associated uncertainty is not adequately gauged. As any over-prediction in system residual life could lead to catastrophic safety hazards, instead of blindly relying on the prediction from black-box models, quantifying the uncertainty in RUL prediction allows maintenance to be scheduled with confidence.

Sources of uncertainty can be categorized as aleatory uncertainty, which is inherent in the natural variability of the physical system, e.g., measurement noise, and epistemic uncertainty, as originating from a lack of knowledge, e.g., model assumption and inaccuracy [19]. In bearing RUL prediction, it is found that the variability of actual bearing lifespans is evident in a few openly available run-to-failure experiments [17,18], even among identical bearing models operating under the same speed and radial load. This variability could be attributed to manufacturing inconsistency, different assembly tolerances, and mounting errors that cause variations in bearing degradation dynamics. Furthermore, since bearing faults may initiate at different times, leading to varying RUL estimates towards failure, a DL model might be challenged by similar input measurements that have different RUL targets, which renders the prediction more difficult at the early fault stage. To alleviate this issue, some research normalizes the RUL target of each data sample based on the bearing’s actual lifespan, which is known from experiments, where a network is trained to predict an RUL percentage ranging from zero to one. However, it should be noted that such a percentage may not provide a practical solution to prognostic applications and might be contradictory when reconstructing the RUL prediction into a physical time scale. Indeed, the trustworthiness of DL-based prediction models is a key prerequisite before pragmatic applications. Beyond the deterministic prediction of RUL, an effective prognosis model should not only forecast the RUL but also assess the associated uncertainty [20,21]. Such quantified uncertainty becomes crucial in the prevention of unexpected failures due to over-prediction and the minimization of costs from early replacements or extra maintenance because of the under-prediction of RUL [19].

To enable probabilistic modelling in DL, one straightforward route would be the generative model that involves learning data distributions, where synthetic data can be generated by sampling from the distributions learned. In this domain, the most compelling advancement might be the variational autoencoder (VAE) that incorporates Bayesian inference into auto-encoders (AE). VAE aims to learn a probabilistic encoding of inputs for generating new synthetic data, such as artificial speech synthesis [22] and image generation [23]. VAE is known as an extension of classical AE but intrinsically differs in its mathematical formulation [24]. By encoding input data as latent distributions rather than a deterministic latent space, VAE is able to capture the principal components and uncertainty within data, facilitating unsupervised learning applications such as noise reduction and anomaly detection [25]. To generalize VAE to supervised learning, VAE for regression (VAER) is innovatively developed for jointly predicting target variables while learning interpretable latent representations [26]. In the field of PHM, VAE has been applied to out-of-distribution detection to identify malfunction in cyber-physical systems [27]. For bearing prognosis, the MLP-based VAER (MLP-VAER) is introduced to RUL prediction using a single-channel vibration measurement [28]. 

Motivated by the demand for interpretability and uncertainty quantification for bearing RUL prediction in plant maintenance, this study attempts to extend the capability of envelope analysis to prognosis beyond fault detection by enabling intelligent RUL prediction with quantified uncertainty using envelope spectra input. Different from the conventional prediction models using numerical indicators or time-domain vibration signals, enhanced envelope spectra are used for their interpretable diagnostic insights of physical defect conditions. To handle the variability within life-cycle data while predicting RUL distributions, the convolutional VAE for regression (CVAER) is innovated by integrating the convolutional VAE with a parallel regressor and a latent generator. The objective function of this formulation is also derived based on the evidence lower bound (ELBO). 

First, to obtain an improved representation of bearing defect conditions, enhanced averaged envelope spectra (AES) are computed based on the segmentation of envelope signals to capture clear fault characteristic frequencies (FCF) [2]. Through spectral inspection on AES, fault initiation in bearings could be detected at the earliest opportunity (for the benefit of benchmarking RUL prediction performance). Different from the conventional practice based on statistical HIs and their first passage time of an alarm limit, AES-based fault detection provides a more reliable alternative to generate the first detection time (FDT) of faults, which ascertains the starting point of bearing degradation and implementation of RUL prediction. By design, CVAER adaptively learns the nonlinear mapping from multiple envelope spectra to RUL distributions and captures both types of uncertainty during bearing degradation. Specifically, the probabilistic regressor predicts not only the mean RUL but also its standard deviation (STD) in a single forward propagation. The predicted RUL is then fed to a latent generator network to regularize the latent representations to be RUL-specific, which further harnesses the interpretability of the prediction model. For performance verification, the CVAER is applied to multi-channel run-to-failure vibration data of 15 test bearings [17]. Each bearing’s RUL is predicted using individual implementation for cross-validation. Given the variability of bearing lifespans, RUL variables are normalized based on the maximum life span within the training set and reconstructed to a physical time scale after prediction. In addition, the advantage of the proposed architecture in learning RUL-specific latent representations is illustrated via t-distributed stochastic neighbour embedding (tSNE). Nonetheless, a comparative and ablation study against MLP, CNN, and MLP-VAER [28], also shows the superiority of CVAER in terms of prediction accuracy. To the best of our knowledge, this study may be the first attempt to introduce a supervised variational autoencoder for regression to realize intelligent RUL prediction with uncertainty quantification. The use of an envelope spectrum is advantageous for the correct detection of bearing defects and then CVAER to estimate RUL just using a few measurements once defects are detected. This is a requirement for industries, and hence supporting maintenance engineers to plan maintenance and remedial action to minimize the maintenance overhead and maintain overall safety. The main contributions of this paper can be summarized as follows: 

(1) The prognosis capability of envelope analysis is enhanced by enabling intelligent RUL prediction from multi-channel envelope spectra input rather than raw vibrations or numerical indicators. (2) The probabilistic CVAER network is developed with ELBO for generative modelling of life-cycle spectral data while providing predictive RUL distributions rather than deterministic estimates. (3) Interpretability of the DL-based RUL prediction method is enhanced thanks to the quantified uncertainty, the RUL-specific latent representation, and the averaged envelope spectra that are physically relevant to bearing defects. (4) Comparative analysis with benchmark models has validated the effectiveness of the proposed method for RUL prediction using envelope spectra, demonstrating its potential for practical maintenance planning. 

The remainder of this work first provides the theoretical backgrounds of VAE to reveal its probabilistic nature in Section 2. The proposed RUL prediction method covering the averaged envelope spectra and CVAER is elaborated on in Section 3. The use of the envelope spectrum makes sure that it contains only bearing-related information by removing other rotor-related frequencies, hence it improves the RUL prediction. Further, applications to experimental studies are presented in Section 4. A brief discussion of the result is delivered in Section 5. Concluding remarks are made in Section 6.

## 2. Preliminaries

### 2.1. VAE

A classical autoencoder (AE) network is characterized by two typical sub-nets including the encoder, ϕ for learning a compressed latent space $z=\phi (X)\in R^{n}$ from l-dimensional data $X\in R^{l}\;(l>n)$ and the decoder, ψ for generating a synthetic representation X′=ψ(z) that approximates the original data X. This procedure is applied in unsupervised learning applications, including but not limited to dimensionality reduction, noise removal, and anomaly detection, such as sparse AE [29] and denoising AE [25,30]. Mathematically, an AE network aims to solve the following minimization problem,

(1) $$\underset{\theta }{\min}{\left\|\psi \left(\phi \left(X\right);\theta \right)-X\right\|}^{2}+\lambda \;\Omega (\phi (X;\theta )),$$

where $\Omega \left(\phi \left(X\right)\right)=\Omega (z)$ denotes the regularization term on latent encoding z that integrates a priori knowledge, e.g., sparsity, λ is a regularization factor, and θ refers to the network parameters. With the encoder–decoder architecture, an AE learns deterministic mappings from input to a latent space that facilitates synthetic reconstruction. 

**Figure 1:**
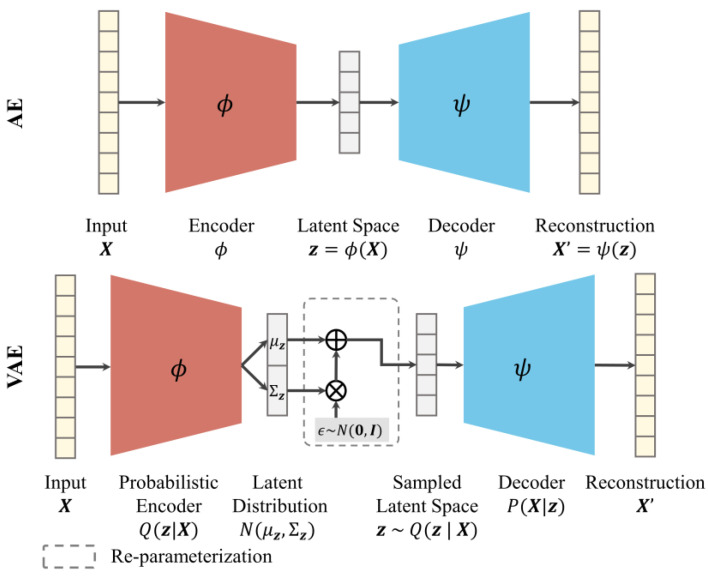
The architecture of AE and VAE networks.

A variational autoencoder (VAE), however, is a generative extension of AE with the capability of probabilistic modelling for generating ‘unseen’ data. Distinctively, VAE is characterized by probabilistic latent space or distributions that allow for data synthesis with more variations by sampling from the distributions learned. Figure 1 compares AE with VAE, where the encoder of VAE learns parameters to characterize a multivariate Gaussian distribution used for sampling probabilistic latent space $z\sim N\left(\mu_{z},\Sigma_{z}\right)$. The decoding from z synthesizes more complex representations that are like X thanks to the introduction of variation$\;\Sigma_{z}$. 

The aim of VAE is to find the optimal model parameters that best describe the distribution of observation data, i.e., maximizing the probability of each sample in the dataset P(X) by probabilistic modelling. Based on Bayes’ theorem [24,31], this can be indirectly achieved by maximizing the likelihood of X conditioned on latent space z, P(X|z), as

(2) P(X)=∫P(X|z)P(z)dz,

where P(z) is a deterministic standard Gaussian prior, i.e., N(0,I) (I denotes an identity matrix) particularly assumed on the latent space in VAE. The conception behind this is that any arbitrarily complex distributions with multiple dimensions can be generated by mapping combinations of Gaussian variables through an adequately complex approximation function [32]. In the context of VAE, this approximation function is realized via layers of neural networks and learned by training the decoder P(X|z). To facilitate the back-propagation of error during the model fitting, the reparameterization trick is typically introduced in VAE by sampling z as $z=\mu_{z}+\epsilon \sqrt{\Sigma_{z}}$, $\epsilon \sim N\left(0,I\right)$ [31], rendering a differentiable network where weights and biases can be efficiently optimized with a stochastic gradient descent algorithm.

### 2.2. Objective of VAE

In maximum likelihood estimation (MLE), the best parameter z that maximizes the probability of observed data P(X|z) is of interest because the integration over z for the marginal likelihood P(X) is intractable. Although it is straightforward to approximate P(X) by sampling a number of latent variables, $z_{i}\;(i=1,2,\;\cdots ,n)$ and calculate the discretized summation, an accurate approximation to P(X) requires a sufficiently large number of n, which is paradoxically in a divergent path against the encoder design attempting to learn a dimension-reduced latent space z. Here, VAE introduces an auxiliary distribution Q(z|X) (encoder) that conditions latent variables z on X for approximating the posterior density function given observation data, P(z|X). Consider the Kullback–Leibler (KL) divergence for measuring the distribution similarity between Q(z|X) and P(z|X),

(3) $$D_{KL}\left[Q\left(z|X\right)\left\|P\left(z|X\right)\right.\right]=\int Q\left(z|X\right)log\frac{Q(z|X)}{P(z|X)}dz=E_{z\sim Q}[\log Q\left(z|X\right)-\log P\left(z|X\right)],$$

which leads to the following by rewriting $P\left(z|X\right)=\frac{P(X|z)P(z)}{P(X)}$,

(4) $$D_{KL}\left[Q\left(z|X\right)\left\|P\left(z|X\right)\right.\;\right]=E_{z\sim Q}[\log Q\left(z|X\right)-\log P\left(X|z\right)-\log P\left(z\right)+\log P(X)],$$

(5) $$\mathrm{ELBO}=\log P(X)-D_{KL}\left[Q\left(z|X\right)\left\|P\left(z|X\right)\right.\right]=\underset{Reconstruction\;loss}{\underbrace{E_{z\sim Q}\left[\log P\left(X|z\right)\right]}}\underset{KL\;loss}{\underbrace{-D_{KL}\left[Q\left(z|X\right)\left\|P\left(z\right)\right.\right]}}$$

Here, $D_{KL}\left[Q\left(z|X\right)\left\|P\left(z|X\right)\right.\right]$ in the left-hand side quantifies the approximation error between the encoder $Q\left(z|X\right)$ and true posterior P(z|X), which tends to diminish when the encoder network learns effective z that accurately recovers the input X. As the KL divergence is a non-negative quantity, the marginal logarithmic likelihood, log⁡P(X), is bounded by the right-hand side terms referred to as the evidence lower bound (ELBO): the difference in the expected logarithmic likelihood $\log P\left(X|z\right)$ and the KL divergence of the encoder distribution $Q\left(z|X\right)$ over the standard Gaussian priors P(z) [24]. The likelihood $P\left(X|z\right)$ is commonly assumed Gaussian conditioned on the normally distributed z in standard VAE and parameterized by the decoder network ψ(z;θ), i.e., $P\left(X|z\right)\sim N(X;\psi \left(z;\theta \right),\;\Sigma I)$ where θ denotes decoder parameters and Σ the variance of reconstruction given z. Hence, the learning objective of VAE for maximizing marginal logarithmic likelihood, equivalent to minimizing −log⁡P(X), can be implemented by minimizing the mean squared error (MSE) that measures the reconstruction loss, ${\left\|X-X\prime \right\|}^{2}$,

(6) $$\log P\left(X|z\right)=-\frac{{\left\|X-\psi \left(z;\theta \right)\right\|}^{2}}{2\;\Sigma }-\log\frac{1}{{\left({\left(2\pi \right)}^{l}\;\Sigma \right)}^{1/2}},$$

together with minimizing $D_{KL}\left[Q\left(z|X\right)\left\|P\left(z\right)\right.\right]$ as a latent space regularization term regarding the similarity of $Q\left(z|X\right)$ to a standard Gaussian. Thanks to the regularization of the encoder distribution, a smooth and evenly distributed latent space z is encouraged to help prevent overfitting [33]. In addition, the latent space with disentangled dimensions also facilitates interpretable representations.

## 3. Proposed Method

### 3.1. Fault Detection Based on Averaged Envelope Spectra

Prediction of bearing RUL well before the end of bearing lifespan or failures allows ample time for maintenance scheduling and proactive interventions to prevent unexpected downtime and systematic failures, which is of practical significance in terms of safety and financial costs. In general, bearing lifespans can be divided into two typical stages by the onset of faults, i.e., the healthy stage and the degradation stage [2,10]. Since RUL depends on the bearing’s current condition, predicting RUL during healthy stages may lack reliable patterns to forecast future degradation or confuse decision-making with a fixed prediction over different elapsed times. Therefore, bearing RUL prediction starts to convey practical meanings and gain usefulness after fault detection. 

Study [34] used numerical methods for bearing fault detection, such as envelope analysis for resonance demodulation, dictionary learning for feature extraction [4], matrix and tensor decomposition for adaptive informative band extraction [35,36], DL-based diagnostics [37], etc. A common practice for identifying anomalies or fault events is to set an empirical threshold (e.g., the 3σ rule) on vibration-based statistical health indicators, whose first passage time signifies bearing degradation and the start of RUL prediction. However, applying such an empirical threshold may not be useful for industrial applications. Since detecting early faults in bearings is crucial for identifying degradation stages, robust fault detection is a prerequisite for the subsequent RUL prediction task. In this study, rather than using bearing HIs, averaged envelope spectra (AES) established in [2] are employed to offer a reliable representation of bearing defect conditions. Envelope analysis is utilized based on rotor-dynamics to identify faults in bearings. When bearing defects develop, transient contacts between defects and rotating components generate cyclic impacts that excite high-frequency impulse responses related to the resonance frequency of the bearing assembly. These cyclic components modulating the high-frequency responses are diagnostically informative for various fault types [38], which are referred to as bearing fault characteristic frequencies (FCF), including fundamental train frequency (FTF), ball spin frequency (BSF), ball-pass frequency at outer-race (BPFO) and ball-pass frequency at inner-race (BPFI). To extract bearing FCF, the averaged envelope spectrum has been proposed to enhance spectral components for bearing fault detection [2,38]. Based on spectral inspection, the onset time or the first detection time (FDT) of fault can be effectively identified from the FCF in an envelope spectrum. 

Given an *N*-point acceleration signal, a high-pass filtration step is first applied to remove the inference of rotor dynamics from a low-frequency range [38]. The envelope of the filtered signal is then obtained based on the Hilbert transform. An AES is computed by averaging the spectra over multiple envelope segments of length m, as given by

(7) $$X_{AES}(f_{k})=E\left\{X\left(f_{k}\right)X^{*}\left(f_{k}\right)\right\}=\frac{\sum_{i=1}^{N_{s}}X_{i}(f_{k})X_{i}^{*}(f_{k})}{N_{s}},$$

where $X_{i}(f_{k})$ and $X_{i}^{*}(f_{k})$, respectively, denote the envelope spectrum of the *i*-th envelope segmentation and its complex conjugate, $X_{AES}(f_{k})$ is the AES based on a total of $N_{s}$ averaging, and $f_{k}\in [0,\frac{f_{s}}{2}]$ ($k=0,1,\;\ldots ,\frac{m}{2}-1$) denotes the frequency index of m-point Fourier transform under the sampling rate $f_{s}$. Based on the average operation, the bearing defect frequencies are enhanced by the suppression of the random components. Compared with the raw vibration signal, AES provides an improved and interpretable representation closely related to actual defect conditions. 

### 3.2. Convolutional VAE for Regression (CVAER): A Probabilistic Model

Envelope analysis is a standard procedure for bearing fault diagnosis because of its efficacy in discriminating defect frequencies, whereas its capability to represent bearing degradation conditions seems underexplored. For this reason, a discernible mapping is assumed between envelope spectra and bearing RUL. Inspired by the VAE for regression (VAER) network that estimates human ages via a single brain image [26], previous work introduces MLP-based VAER (MLP-VAER) to bearing prognosis using a single-channel vibration measurement [28]. In order to support maintenance engineers in scheduling timely interventions, it is crucial to accurately estimate bearing life as soon as defects are detected, even using limited measurements. Distinctively, this study utilizes two-dimensional convolutional layers to formulate a convolutional VAER (CVAER) variant that adapts to multi-channel envelope spectra input for statistical prediction of bearing RUL. 

Consider envelope spectral data from continuous vibration measurements, $X=\left\{X^{\left(1\right)},\;X^{\left(2\right)},\;\cdots ,\;X^{\left(T\right)}\right\}$, each $X^{\left(t\right)}\in R^{L\times c}$ denotes c columns of L-point AES dependent on the number of utilized accelerometers, and the time stamp t=1, 2, ⋯, T represents the time step since FDT, i.e., after a bearing fault is detected. During the degradation stage, each $X^{\left(t\right)}$ is correspondent to a target RUL variable $y^{\left(t\right)}\in R^{1}$ denoting the residual time towards the end of life. For one test bearing, T samples of multi-channel AES data have their corresponding RUL label set, $Y=\left\{y^{\left(1\right)},\;y^{\left(2\right)},\;\cdots ,\;y^{\left(T\right)}\right\}.$

**Figure 2:**
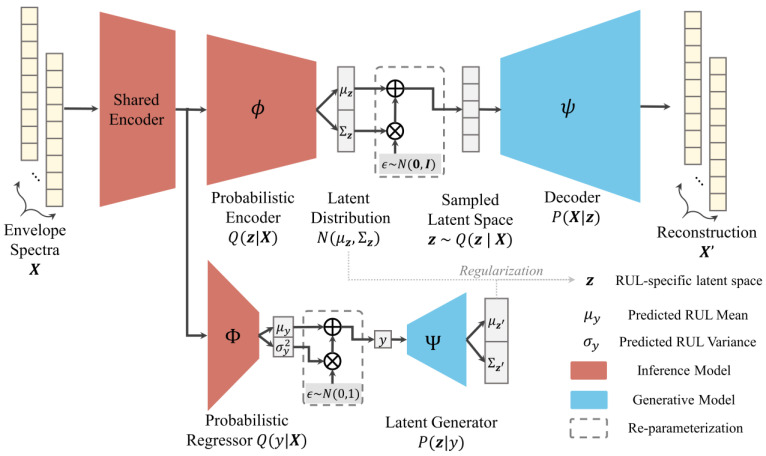
Schematic diagram of the probabilistic CVAER model for RUL prediction.

The CVAER network is designed using a convolutional encoder–decoder structure with an additional regressor and a latent generator specially crafted for the RUL prediction task. For illustration, the schematic diagram of CVAER network is shown in Figure 2. Different from the standard VAE that is commonly used for generative modelling in an unsupervised setting, CVAER inherits the merits of VAE in preventing overfitting while realizing nonlinear mapping between envelope spectra and RUL distributions by supervised learning. 

The establishment of CVAER involves the following ideas about statistical RUL regression and learning meaningful latent space. First, to model the variations in AES during bearing degradation, it is assumed that at a specific time *t* after a bearing fault is detected, a multi-channel AES matrix X is correlated with a latent representation, $z\in R^{n}$, as can be obtained in a standard VAE setting. Second, rather than using a standard Gaussian prior, P(z), z is explicitly conditioned with the corresponding RUL variable y with a prior distribution, P(y) and is thus driven to learn interpretable representations. To achieve this, a regression block, or regressor network, is introduced to predict the RUL target y, which is subsequently utilized by the probabilistic generator to obtain the RUL-specific prior $P\left(z|y\right)$. Hence, denoting the generative model of the envelope spectral data as $P\left(X,z,y\right)$, the marginal likelihood of X can be written as $P\left(X\right)=\int P\left(X,z,y\right)\;dzdy=\int P\left(X|z\right)P\left(z|y\right)P\left(y\right)\;dzdy$.

Specifically, the probabilistic encoder, ϕ is employed to map envelope spectra X to a compressed latent distribution parameterized by a multivariate Gaussian density with mean $u_{z}$ and logarithmic variance $\log\Sigma_{z}$, i.e., $Q(z|X)\sim N(z;f_{\phi }(X;\theta_{\phi }),{g_{\phi }(X;\theta_{\phi })}^{2}I)$, where $f_{\phi }$ and $g_{\phi }$, respectively, denote the encoder network layers with parameters $\theta_{\phi }$ for learning the mean or variance. For the reconstruction of the synthetic envelope spectra X′, the decoder network ψ attempts to generate a representation that assembles X based on the latent space z sampled from Q(z|X) using the reparameterization trick, i.e., $z=\mu_{z}+\epsilon \sqrt{\Sigma_{z}}$, $\epsilon \sim N\left(0,I\right)$. The decoder distribution can then be parameterized as $P\left(X|z\right)\sim N(X;f_{\psi }(z;\theta_{\psi }),\;{g_{\psi }(z;\theta_{\psi })}^{2}I)$. The encoder–decoder network learns a compressed latent representation of AES, which can be further used in the generation of synthetic envelope spectra in data-unbalanced scenarios. Due to the scarcity of trainable data, the latent mapping learned by deep neural nets tends to over-fit and not necessarily relate with the bearing degradation characteristics. To predict of RUL distribution based on envelope spectral input and extract RUL-specific latent representations, the probabilistic regressor and the latent generator are further constructed in CVAER.

To this end, the shared encoder distinctively branches into the probabilistic encoder with the probabilistic regressor, Φ as a univariate normal density Q(y|X) for estimating the RUL target y and its uncertainty, i.e., $Q(y|X)\sim N(y;f_{\Phi }(X;\theta_{\Phi }),g_{\Phi }(X;\theta_{\Phi }))$, where $f_{\Phi }$ and *g*, respectively, denote the inference networks with convolutional layers (parameterized by $\theta_{\Phi }$) for the estimation of RUL mean $\mu_{y}$ and variance $\sigma_{y}^{2}$. The shared encoder design provides a unified representation for capturing essential features from AES input, ensuring both the probabilistic encoder and RUL regressor operate on a consistent feature space. 

In order for the encoder to learn interpretable latent representations, z is regularized by a latent generator network, Ψ that explicitly conditions z on the RUL target y, leading to a conditional RUL-specific prior P(z|y). In other words, given the RUL predictions from the regressor outputs, RUL-specific latent representations can be sampled from the generator distribution, as parameterized by $P(z|y)\sim N(z;u^{T}y,\;\sigma^{2}I)$, where u is a unit-length feature vector related to the bearing RUL characteristics with disentangled dimensions [23]. This implies that the change in latent representations due to estimated RUL does not affect the other latent dimensions (features). Such disentangled representations are favoured in generative modelling as they allow the interpretation of a specific feature and facilitate innovative configurations of other features [23]. For this purpose, the difference between the encoder distribution Q(z|X) and the latent generator P(z|y) is regularized by minimizing the KL divergence. For reference, the probabilistic characteristics of the networks are summarized in Table 1.

### 3.3. Network Architecture

**Figure 3:**
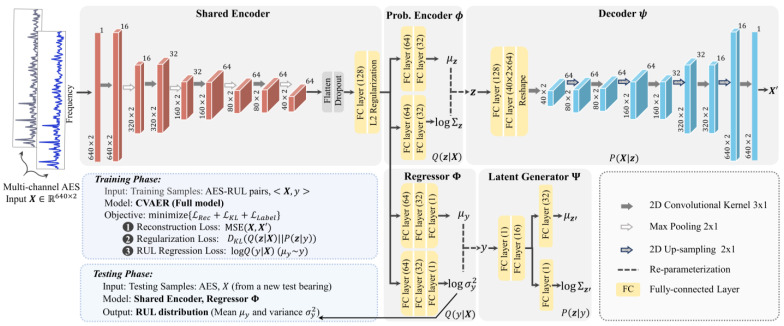
Network architecture of the CVAER.

Convolutional neural networks (CNNs) excel at learning localized features from structured data based on parameter sharing and sparse neuron connectivity. To adapt two-dimensional (2D) envelope spectral input ***X*** from multi-channel vibration measurements, CVAER uses 2D convolutional layers as building blocks for the shared encoder and the decoder network. The parallel probabilistic regressor and latent generator are constructed for RUL prediction with uncertainty estimates and learning interpretable latent space. Figure 3 details the architecture of CVAER network, where two-channel AES input $X\in R^{640\times 2}$ is used as an instance for consistency and will be further explained in the experiment section.

For the extraction of the localized feature within the envelope spectra, the convolutional kernel of size 3×1 is used with rectified linear unit activation (ReLu) for learning nonlinearity mappings. In the shared encoder network, each convolutional layer is followed by a maxpooling layer of size 2 × 1 for dimensionality reduction. The extracted features are fully connected (FC) into intermediate layers with hyperbolic tangent activation (Tanh) to learn probabilistic latent representations. Correspondingly, in the decoder network, a 2D upsampling layer of size 2×1 is utilized after each convolutional layer, such that the reconstruction of synthetic envelope spectra X′ retains consistent dimensions. As denoted on the top of the convolutional layers, the number of kernels for feature extraction is selected to maintain the symmetry of the encoder and decoder networks.

To achieve synchronous extraction of latent distributions $N(\mu_{z},\Sigma_{z})$ and prediction of bearing RUL $N(\mu_{y},\sigma_{y}^{2})$ from spectral input, the initial layers of the convolutional encoder are shared by the probabilistic encoder and the probabilistic regressor. ϕ, Φ, and Ψ are constructed using fully connected layers (dense layers). To prevent overfitting, the dropout regularization and the L2-norm regularization [26] are leveraged at the connection of the shared encoder and the probabilistic encoder. 

### 3.4. Model Fitting: Objective Function of CVAER

The objective of CVAER is to fit probabilistic distributions that are conditioned on bearing RULs while best depicting an envelope spectral dataset X as a bearing degrades. Similarly to VAE, the model parameters of CVAER can be optimized by maximum likelihood estimation (MLE), which indirectly maximizes the evidence, or equivalently the sum of the logarithmic marginal likelihood, $\sum_{t}\log P(X^{\left(t\right)})$. For maximization of log⁡P(X), the auxiliary function Q(z,y|X) is introduced to approximate the posterior density function P(z,y|X) in CVAER, which gives

(8) $$\log P\left(X\right)=\log P(X)\iint Q(z,y|X)dzdy=\iint Q\left(z,y|X\right)\log P\left(X\right)dzdy.\;$$

Based on Bayes’ theorem, inserting $P(X)=\frac{P(z,y,\;X)}{P(z,y|X)}$ and introducing the auxiliary function Q(z,y|X), Equation (8) further writes

(9) $$\log P\left(X\right)=\iint Q\left(z,y|X\right)\log\frac{P(z,y,\;X)}{P(z,y|X)}dzdy=\iint Q\left(z,y|X\right)\log\frac{P\left(z,\;y,\;X\right)\;Q(z,y|X)}{P(z,y|X)\;Q(z,y|X)}dzdy,$$

and

(10) $$L\left(X\right)=\log P\left(X\right)-D_{KL}[Q(z,y|X)||P\left(z,y|X\right)]=E_{Q}\left[\log P\left(X,z,y\right)-\log Q\left(z,y|X\right)\right],$$

where L(X) denotes the ELBO of CVAER ensured by the nonnegativity of the KL divergence. The auxiliary distribution Q(z,y|X) can be further factorized as $Q\left(z,y|X\right)=Q\left(z|X\right)Q\left(y|X\right)$ based on the mean-field theory, where the latent variables z and the RUL target y are assumed to be conditionally independent [26]. In addition, the RUL target y in the CVAER is assumed to be disentangled from the latent variables, such that $P\left(X|z,\;y\right)=P\left(X|z\right)$. Hence, the objective function of the CVAER network based on MLE can be further rewritten as

(11) $$L\left(X\right)=\iint Q\left(z,y|X\right)\log\frac{P\left(X,z,\;y\right)}{\;Q\left(z,y|X\right)}dzdy=\iint Q\left(z,y|X\right)\log\frac{P\left(X|z,\;y\right)P\left(z|y\right)P\left(y\right)\;}{\;Q\left(z,y|X\right)}dzdy\;=\iint Q\left(z,y|X\right)\log\left\{P\left(X|z\right)\cdot \frac{P\left(z|y\right)\;}{Q(z|X)}\cdot \frac{P\left(y\right)\;}{Q(y|X)}\right\}dzdy=-\left\{L_{Rec}\left(X\right)+L_{KL}\left(X\right)+L_{Label}\left(X\right)\right\},$$

which is essentially minimizing three loss terms, i.e., the reconstruction loss term,

(12) $$L_{Rec}\left(X\right)=-\iint Q\left(z,y|X\right)\mathrm{logP}\left(X|z\right)dzdy=-\iint Q\left(y|X\right)dyQ\left(z|X\right)\mathrm{logP}\left(X|z\right)dz\;\;=-\int Q\left(z|X\right)\log P\left(X|z\right)dz=-E_{Q(z|X)}[logP\left(X|z\right)],$$

the KL divergence term (for regularizing latent distributions),

(13) $$L_{KL}\left(X\right)=-\int Q\left(y|X\right)\left\{\int Q\left(z|X\right)\log\frac{P\left(z|y\right)\;}{Q(z|X)}dz\right\}dy=E_{Q(y|X)}\left[D_{KL}\left[Q\left(z|X\right)||P\left(z|y\right)\right]\right],$$

and the RUL label loss term (for RUL regression),

(14) $$L_{Label}\left(X\right)=-\int Q\left(y|X\right)\log\left\{\frac{P\left(y\right)\;}{Q(y|X)}\right\}dy=D_{KL}\left[Q\left(y|X\right)||P\left(y\right)\right],$$

During the training phase, the AES samples with the according RUL labels are fed to the CVAER model for learning the network parameters that jointly optimize the three above loss terms. It is noted that $L_{Rec}$ and $L_{KL}$ involve the expectation with respect to the posterior distributions, instead of direct computation, the stochastic gradient variational Bayes estimator (SGVB) is applied to provide a differentiable objective function and enable model training via back-propagation [31]. Specifically, the minimization of $L_{Rec}$ and $L_{KL}$ can be approximated by minimizing the reconstruction loss via MSE and minimizing the KL divergence between two Gaussian distributions. For the fitting of RUL prediction, the minimization of $L_{Label}$ is equivalent to minimizing $E[logQ\left(y|X\right)]$ as the RUL labels y of the spectral data are known as ground-truth during training.

### 3.5. RUL Prediction: Testing the CVAER Model

During the testing phase, given the envelope spectra of a new test bearing at its degradation stage, only the encoder and regressor networks are utilized to predict the RUL. Thanks to the probabilistic regressor, the RUL distribution can be predicted under the Gaussian assumption with the estimated mean and STD. The uncertainty of the RUL prediction at test time is quantified based on the variance of the envelope spectra of the training samples and the regularization from the KL loss term $L_{KL}$ for learning the RUL-specific latent representations from the available AES data during model fitting. 

Apart from the prediction of RUL, for the generation synthetic envelope spectrum, one could directly sample the latent space z from the standard multivariate Gaussian N(0, I) and feed to the decoder network. The synthetic AES with controllable variations can be generated to augment bearing degradation data.

## 4. Experimental Studies and Results

### 4.1. Experimental Settings

#### 4.1.1. Mechanical Setup

**Figure 4:**
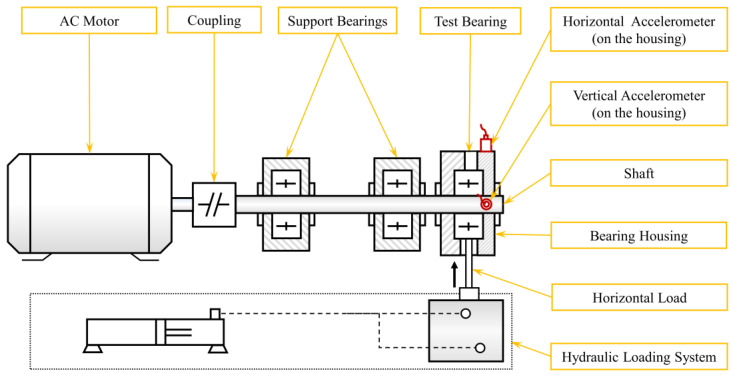
The schematic diagram of the bearing test rig for run-to-failure experiments.

To verify the prognosis performance, the CVAER model is applied to bearing RUL prediction using historical vibration measurements from the accelerated run-to-failure experiments. The bearing degradation data provided by Xi’an Jiaotong University and Sumyoung Technology, known as XJTU-SY datasets [17], are selected for the coverage of multiple operating conditions and different bearing failure modes. For illustration, Figure 4 presents the setup of the experimental rig, which consists of an induction motor with speed control, a flexible coupling, a shaft, two support bearings, and a test bearing (LDK UER204). The radial load of the motor rotor-bearing system is imposed on the test bearing housing from the horizontal direction with a hydraulic loading system. The specifications of the test bearing are summarized in Table 2.

#### 4.1.2. Vibration Sensors and Measurement Scheme

To acquire bearing life-cycle vibration data, two PCB 352C33 accelerometers were mounted on the test bearing housing in orthogonal directions [17]. Note that the selected accelerometers have a natural frequency of above 50 kHz, providing an effective measurement frequency range covering bearing resonance to ensure fault detection reliability. Detailed performances of the accelerometer are presented in Table 3.

In the experiments, run-to-failure tests were conducted for 15 test bearings with different speed-loading combinations, which ended up with various failure modes. For each test, bearing vibration signals were cyclically sampled using DT9837 dynamic data acquisition module at a fixed interval. Vibration data were monitored from the healthy stage to the predetermined failure threshold, i.e., ten times the maximum vibration acceleration amplitude under the healthy condition [17]. The details of all available data subsets are summarized in Table 4.

### 4.2. Bearing Fault Detection via AES

**Figure 5:**
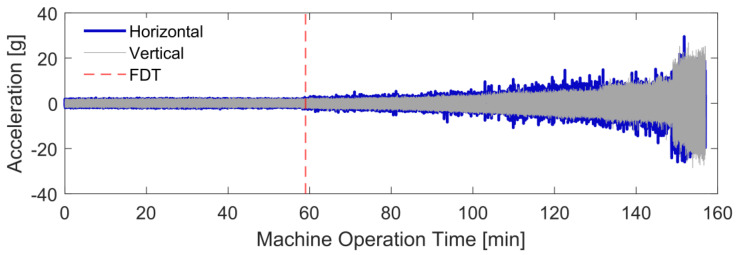
Raw vibration acceleration signals of Bearing 1–3.

**Figure 6:**
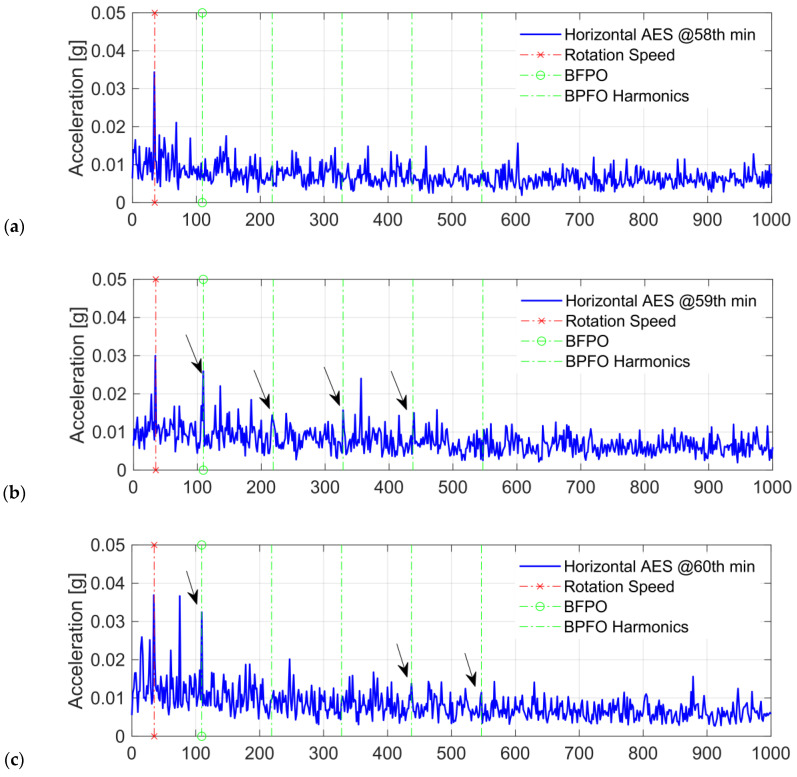

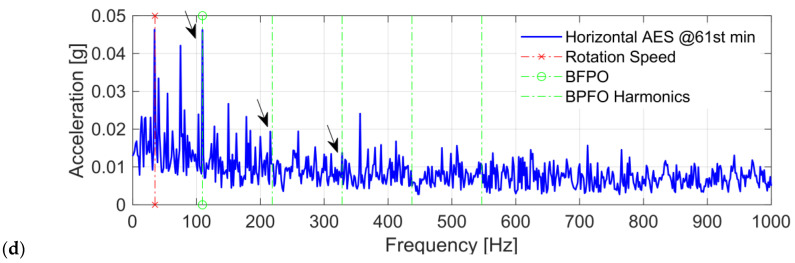
The AES around fault detection time. (**a**–**d**): Horizontal AES of Bearing 1–3 from the 58th to 61st minute. The outer-race fault components, BPFO and its harmonics, are observed from the 59th minute, indicating the initiation of the outer-race fault.

First, the AES for the two-channel acceleration measurements is computed to detect early-stage faults, which helps identify FDT and the bearing degradation stage. The segmentation length used for the AES is m=16384 with the overlap rate providing 200 segments for averaging. The vibration signals of the test bearing ‘Bearing 1–3’ are shown in Figure 5. Due to the hydraulic loading direction, vibration accelerations from the horizontal channel have relatively higher amplitude. Based on envelope analysis, informative defect frequencies can be demodulated from the cyclic bearing resonance responses, which is known as resonance demodulation. From the envelope spectrum, bearing defects can be detected by identifying distinct frequency peaks and modulation patterns matching bearing FCFs and their harmonics. According to the bearing dimensions and the operating speed, the theoretical BPFO for Bearing 1–3 is 107.91 Hz [17]. By spectral inspection of the AES for all the experimental operation time, BPFO and its harmonics can be identified from the horizontal AES after the 59th minute, indicating that the outer-race fault has developed since this time. This can be confirmed by the continuous appearance of BPFO harmonics in the horizontal AES from Figure 6b–d, except Figure 6a. 

#### 4.2.1. Variation in AES Across Bearing Lifetime

To observe the changes in the AES from healthy condition to degradation, the AES of both accelerometer channels are gathered from each operation time step to generate historical spectral contour plots. For illustration, the spectral contour plots, as the characteristic ‘operation time to spectral frequency’ representations, are shown for the horizontal vibration acceleration of ‘Bearing 1–3’ and ‘Bearing ‘3–1’.

**Figure 7:**
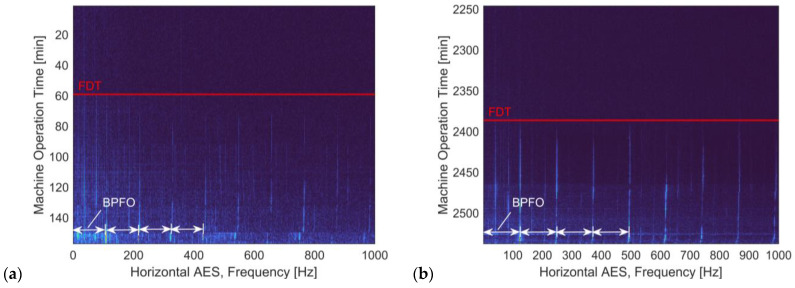
The horizontal AES contours with respect to machine operation time for (**a**) Bearing 1–3 and (**b**) Bearing 3–1. The onsets of the BPFO components are, respectively, identified as the 59th minute and the 2386th minute, as indicated by the red solid lines.

From Figure 7, it is observed that the outer-race fault component and its higher harmonics become more intense as the run-to-failure experiment proceeds. In addition, the harmonic patterns of the BPFO component are increasingly distinctive at the latter stage of the experiment, as the test bearing approaches the end of life. However, the development behaviour of the higher order defect frequencies is hardly straightforward. It is noticed from both run-to-failure processes that different orders of the BPFO harmonics gain their dominance at different times along the bearing degradation, which implies the increment of fault signatures in the envelope spectra may be nonlinear. 

On this basis, it is assumed that complex and nonlinear degradation information lies in the variation in the AES during the bearing degradation stage, which motivates the introduction of the deep learning model based on CVAER for the generative modelling of AES and the probabilistic RUL prediction. 

#### 4.2.2. First Detection Time and Ground-Truth RUL

Based on the spectral inspection on AES, the fault initiation of the test bearing is identified to determine the first detection time (FDT) of fault, which marks the beginning of bearing degradation. In addition, with the FDT carefully determined by enhanced envelope analysis, the performance of different RUL prediction models can be reasonably compared based on controlled degradation periods. As a reference, Table 5 summarizes the FDTs of all test bearings based on the fault detection by spectral inspection on AES.

**Figure 8:**
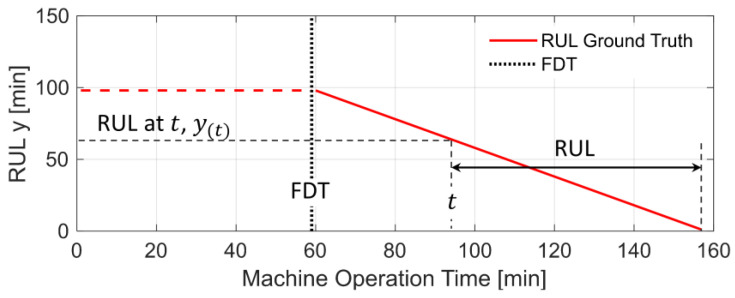
The generation of the RUL targets of ground truth (illustrated via Bearing 1–3).

Next, based on the piece-wise linear RUL model [10], the RUL target y of ground truth can be computed given the full lifespan of each test bearing, where the RUL during the bearing degradation is used for fitting the prediction model, as illustrated with Bearing 1–3 as an example in Figure 8.

Data normalization is a standard procedure in the data pre-processing stage of ML applications. Normalizing data to standardized centre or zero centre helps accelerate the convergence of gradient descent in optimization. Similarly, to prevent instability in fitting neural networks, the RUL label of input data should be normalized to a reasonable range. Existing RUL prediction research normalizes the RUL labels of each bearing into the range from 0 to 1 based on the current time $T_{t}$ and the priorly known end of life time $T_{e}$ from experiment, such as ${\tilde{y}}_{\left(t\right)}=\frac{{RUL}_{\left(t\right)}}{T_{e}-FDT}=1-\frac{T_{t}-FDT}{T_{e}-FDT}$ [16]. Despite the accuracy achieved, it is noted that such normalization may lead to confusion in practical prognosis application, where the RUL in physical time scale is preferred rather than a percentage of life. 

Thus, this study performs normalization based on the available data for training, i.e., RUL labels of each bearing are normalized as $\hat{y}$ using the minimum and maximum RUL values for all the bearings in the same training set S and validation set V,

(15) $$\hat{y}=\frac{y-\underset{y}{\min}\left(S+V\right)}{\underset{y}{\max}\left(S+V\right)-\underset{y}{\min}\left(S+V\right)},$$

During the testing phase, the actual bearing RUL could be recovered to a practical time scale using the above equation, which is free from introducing the end-of-life time prior to the testing data into training. To mitigate computational burden, instead of fitting the full-length envelope spectra, the frequency range from 0 to 1 kHz is segmented for training, which leads to the AES input with 640 frequency bins, i.e., $L=1000/f_{s}*m=640$. For the AES of at time *t*, $X^{\left(t\right)}\in R^{L\times c}$, the probabilistic regressor of CVAER is established to adaptively learn the latent mapping, $\Phi (x)\;:\;X^{\left(t\right)}\in R^{L\times c}\to {\hat{y}}_{\left(t\right)}\in R^{1}$, for predicting the normalized RUL directly from the envelope spectra. Thus, at each time step, the two-channel AES input, $X^{\left(t\right)}\in R^{640\times 2}$, and the corresponding RUL label ${\hat{y}}_{\left(t\right)}$, denoted by the AES-RUL pair, $<X^{\left(t\right)},{\hat{y}}_{\left(t\right)}>$ make one training sample during the model fitting.

### 4.3. Cross Validation Settings

To verify the performance of the CVAER model for bearing RUL prediction, multi-channel envelope spectral data are prepared based on a condition-specific mechanism for cross-validation: The AES-RUL samples of each test bearing are used as the testing set T, leaving the samples from the other bearings in the same operating condition as the training and validation set. Specifically, the division of the training set to the validation is 70% to 30%. In total, 15 implementations of the CVAER network are conducted across the three working conditions, each with five individual test bearings. 

As can be noticed from the experiment results, the degradation-stage lifetimes (or the RUL since FDT) of the same bearing models under identical operating conditions end up in a varying range, revealing the fact that the degradation rates differ even when the speed and loading are controlled. That is, different test bearings have different RULs at the same prediction time since FDT. As a result, the latent mapping from the input to the RUL tends to be nonlinear and non-injective. To confront the challenge, apart from normalizing RUL labels, the training samples are balanced with the weighted training strategy [39]: In the same operating condition, the training samples from the bearings with the longest or shortest lifespans are assigned with fewer weights by repeating the training samples from the bearings of moderate lifespans. In this way, the influence of the bearings with extreme lifespans is appropriately controlled during model fitting. For reference, the data settings used for the 15 cross-validation groups are summarized in Table 6.

During model fitting, the adaptive moments (ADAM) optimizer is employed with a mini-batch gradient descent scheme using an initial learning rate of 10^−3^. For each data setting, the CVAER network is implemented with a mini-batch size of 64 training samples with the maximum iteration number at 500 with an early stopping strategy. Based on the objective function of CVAER involving three loss terms, the validation set is employed for tuning network hyper-parameters, evaluating model performance, and determining the end of training. In each implementation, the weights after model fitting are reserved for testing RUL prediction accuracy and generalization performance. In sum, the CVAER network was developed using TensorFlow 2.7 in Python 3.7.3. The implementations were carried out on the high-performance computing cluster at the University of Manchester using eight CPU cores and two Tesla V100 GPUs with a total of 32 GB graphics memory. A summary of the algorithm is provided in Appendix A Algorithm A1.

### 4.4. RUL Prediction Results

**Figure 9:**
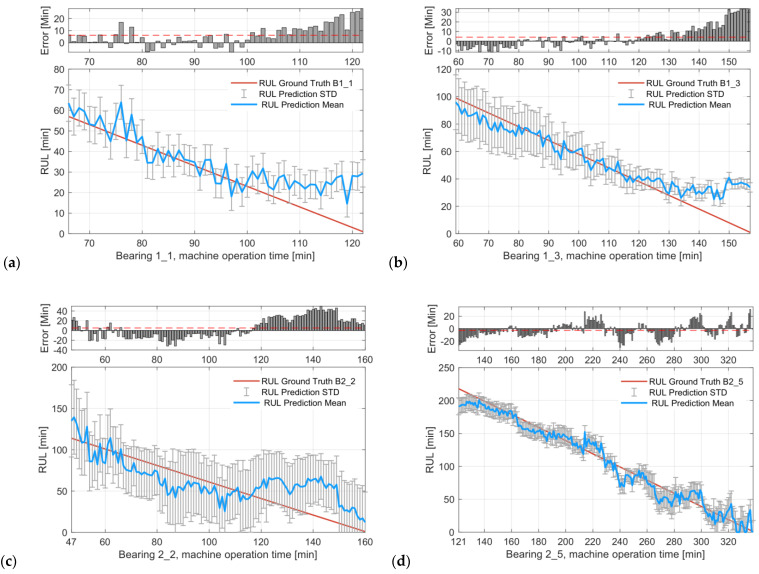

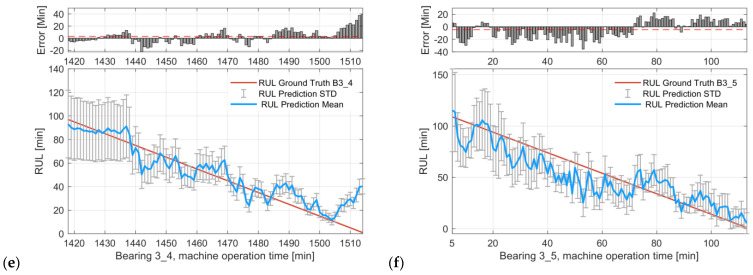
RUL prediction results based on CVAER at the bearing degradation stage for (**a**) Bearing 1–1, (**b**) Bearing 1–3, (**c**) Bearing 2–2, (**d**) Bearing 2–5, (**e**) Bearing 3–4, and (**f**) Bearing 3–5. The mean predicted RULs for the test bearings are shown in blue solid lines with the standard deviation (STD). The error bar plots present the prediction error between the mean prediction and the RUL of ground truth (in red solid line).

At the testing stage, the learned CVAER model for each cross-validation group is tested using the testing bearing samples specified in Table 6. The RUL prediction results, including mean predicted RUL and its standard deviation, are reconstructed into a physical time scale (in minutes) via Equation (15). For illustration, the results of the RUL prediction across three different operating conditions are shown in Figure 9, where the predictions are provided as Gaussian distributions for uncertainty estimation with the prediction error bars.

The results show that the CVAER model captures the decreasing trend in the predicted RUL since the FDT. The mean RUL predictions (in blue solid lines) closely follow the RUL of ground-truth (in red solid lines). The bar plot on the top of the prediction is given by the difference between the mean prediction against the RUL of ground-truth, wherein a positive error represents an over-prediction while a negative error indicates an under- or conservative prediction of RUL. For example, as in Figure 9a, accurate RUL predictions are obtained for Bearing 1–1 at the initial degradation stage since the 66th minute, with the average prediction error at 6.22 min (denoted by a red dashed line). Specifically, at the 70th minute, the bearing RUL predicted by the CVAER is 53 min, which is the same as the ground-truth RUL of Bearing 1–1. This suggests that the proposed model could provide accurate RUL estimations directly from the averaged envelope spectra given the measured vibration signals. Other examples including Bearing 1–3, Bearing 2–5, and Bearing 3–4 also show accurate RUL predictions at the vicinity of FDT, which indicates the effectiveness of RUL prediction by the proposed method even at the early stage of bearing faults. 

Apart from the mean predicted RUL, the uncertainty of the prognosis prediction is evaluated by the standard deviations (STD), as shown along with the estimated RUL, which is facilitated by the inference model of the probabilistic regressor in CVAER. Based on the empirical 3σ rule, the RUL predictions within one STD of the mean represent about 68.27% of all possible predictions given by the normal distribution. A two-sigma (or two-STD) interval around the mean indicates that around 95.45% of the RUL estimation is likely to fall within this range, which evaluates the confidence level of the RUL prediction and guides further prognostic decision-making. From Figure 9, it is observed that the predicted STD intervals well cover the RUL of ground-truth. In addition, it is seen from the results of Bearing 1–3, 2–2, 3–4, and 3–5 that the STD of the predictions are initially large and diminish as time progresses. This reflects the fact that the early-stage uncertainty in the RUL prediction is likely larger due to the weak intensity of the incipient faults and the variability of the defect mechanism. As the bearing approaches failures, the fault and degradation features within the envelope spectra become more evident, leading to less STD and increased confidence in the RUL prediction by CVAER.

**Figure 10:**
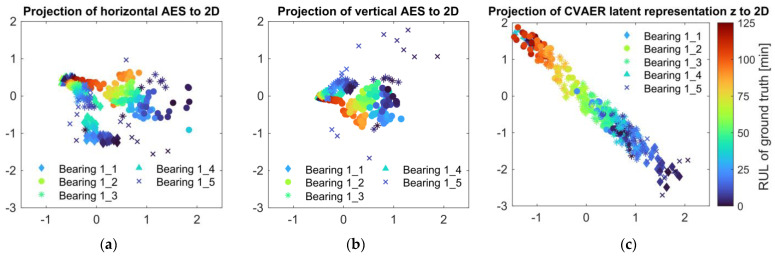
The 2D embedding based on t–SNE using the envelope spectra of the test bearings under Condition 1. The projection of (**a**) the AES from the horizontal channel into a 2D plane, and (**b**) the AES from the vertical channel into a 2D plane. (**c**) The projection of the latent representations learned by the CVAER using the network parameters from Group 3.

To demonstrate the feature extraction performance of the CVAER model, the t-distributed stochastic neighbour embedding (tSNE) method is further utilized to visualize the latent representations of the AES learned via the encoder network of the CVAER. The tSNE method is essentially a dimensionality reduction tool for visualizing high-dimensional data in a low-dimensional representation by optimizing the t-distribution similarity to maintain the local structure. For the test bearings under Condition 1, a total of 382 envelope spectral samples are gathered as $X_{C1}\in R^{382\times 640\times 2}$, where the horizontal-channel AES $X_{C1}^{\left(H\right)}\in R^{382\times 640}$ and the vertical-channel AES $X_{C1}^{\left(V\right)}\in R^{382\times 640}$ are, respectively, mapped into a 2D embedding $P_{X}^{\left(H\right)}\in R^{382\times 2}$ and $P_{X}^{\left(H\right)}\in R^{382\times 2}$, as shown in Figure 10a,b. Among different test bearings, although the AES samples with higher RUL labels tend to cluster, both the projections of the raw AES show random or unstable patterns with respect to the corresponding RUL. 

Based on the CVAER model, the latent representations $z\in R^{382\times 32}$ of the AES samples in Condition 1 ($X_{C1}$) are first obtained using the network parameters learned from the previous implementation. Then, the latent representations are projected into the 2D plane $P_{z}\in R^{382\times 2}$ as shown in Figure 10c. By contrast, a distinct directional pattern is formed among these latent samples with their corresponding RUL in a typical order (as indicated by marker colour). This suggests that from the envelope spectral input $X_{C1}\;$to z, the CVAER network could effectively learn RUL-specific latent representations that are closely aligned with the bearing degradation process, meanwhile enhancing the prediction of bearing RUL.

With the proposed method, averaged envelope spectra serve to not only extract fault diagnostic information but also pave the way for bearing fault prognosis by facilitating the prediction of RUL distributions. Based on the above results, in addition to the well-acknowledged diagnostic performance, the prognostic potential of the envelope analysis is also harnessed through the CVAER model.

### 4.5. Comparison and Ablation Study

For performance verification, comparisons are made against three backbone RUL prediction models based on either one- or two-channel AES input, where each model lays the foundation and motivates the proposed CVAER. For two-channel AES input, the 2D convolutional neural network (CNN) model is constructed to mimic the encoder–regressor architecture in CVAER. For the comparison over the models using only single-channel (i.e., the horizontal-channel) AES input, the VAE regression model based on multi-layer perceptron (MLP-VAER), and the classical multi-layer perceptron (MLP) model with five fully connected layers is employed for benchmarking. Similarly, the structure of the MLP model emulates the design of the encoder–regressor in the MLP-VAER.

**Figure 11:**
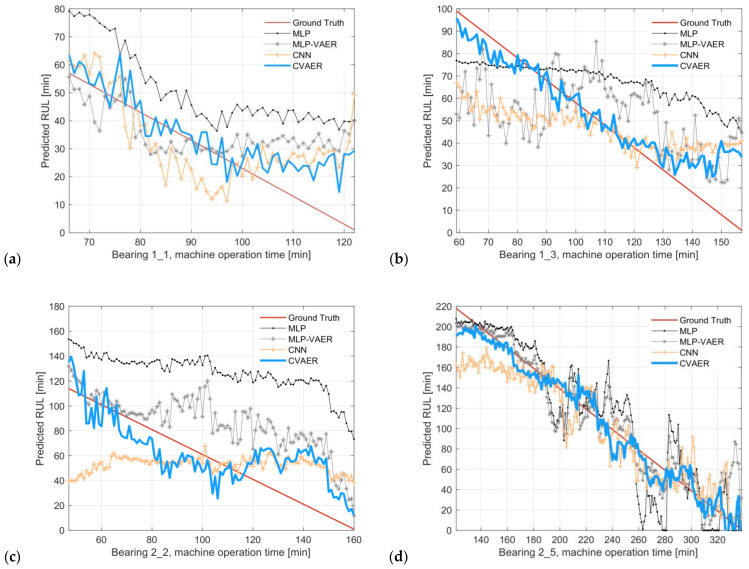

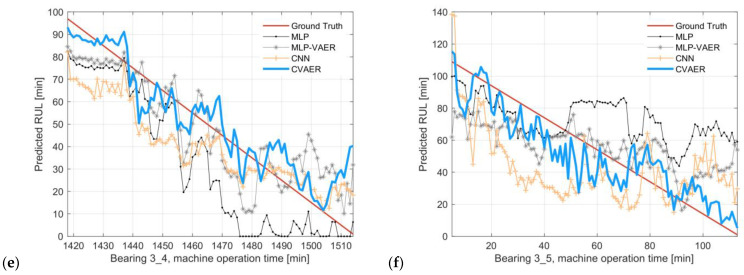
RUL prediction results of CVAER with comparisons against other benchmark models. (**a**) Bearing 1–1, (**b**) Bearing 1–3, (**c**) Bearing 2–2, (**d**) Bearing 2–5, (**e**) Bearing 3–4, and (**f**) Bearing 3–5.

For each model, all the cross-validation groups are implemented with the respective data settings in Table 6, and the results of (mean) RUL prediction across three operating conditions are presented in Figure 11, where the blue solid lines mark the predicted RULs via the proposed CVAER model. 

It is observed that the CVAER outperforms other benchmarks in capturing a decreasing trend in the predicted RUL, which closely follows the RUL of ground-truth. Take the result of Bearing 1–1 in Figure 11a as an example, the predicted RUL using MLP deviates from the ground-truth by an over-prediction of about 20 min, while the MLP-VAER model improves the prediction performance and obtains accurate estimates around the 73rd and the 90th minute during the experiment. This improvement indicates the benefit of the VAER model for performing regression tasks while learning a disentangled latent distribution conditioned on the target variable, which aligns with the conclusion obtained by the previous research [26]. In Figure 11a, the CNN takes two-channel AES input and provides better predictions than the MLP, especially at the early stage of the experiment around the 73rd minute. For the proposed CVAER model, the RUL prediction performance at the early degradation stage is further improved. 

Besides Bearing 1–1, for the test bearings under other operating conditions, the CVAER model also provides more accurate RUL predictions than the other competing networks, as shown in Figure 11b–f. This indicates the performance increment in the CVAER by taking advantage from both CNN and VAER.

For the quantitative assessment of the prognosis performance, the accuracy of the RUL prediction is measured using the root-mean-square error (RMSE) between the reconstructed (mean) RUL prediction and the RUL of ground-truth at the bearing degradation stage. The accuracy metrics of all 15 cross-validation groups implemented using different network models are summarized in Table 7, where the bold font denotes the best accuracy with the lowest RMSE and the ‘*’ signs the runner-up results.

Overall, the CVAER is the most accurate model in terms of the prediction error, yielding an average RSME of 28.47 min. Specifically, for Bearing 1–1, the prediction RMSE of CVAER is 10.48 min, which is approximately half that of the MLP. For Bearing 2–3, despite the higher prediction error due to the significant difference in the lifespans of the test bearings under operating Condition 2, CVAER still demonstrates the lowest RMSE by contrast to other network models.

On the one hand, the CVAER and CNN exhibit the two lowest average RMSE values, which suggests that the RUL prediction using both the horizontal- and vertical-channel AES generally outperforms the prediction solely based on the horizontal-channel AES. On the other hand, the outperformance of CVAER over the CNN model highlights that the combination of VAE for learning the RUL-specific latent distributions could effectively regularize the probabilistic regressor network in predicting bearing RUL. The improvement brought by the VAE regression model is further supported by the lower average error achieved by MLP-VAER compared to MLP. 

Moreover, in the comparison between CNN and MLP-VAER, it is seen that CNN is superior in some implementation groups while MLP-VAER outperforms in others. Compared to the MLP, both models obtain superior prediction accuracy with close average RMSE over all cross-validation groups, which indicates the incremental performance provided by the VAER and CNN. These performance gains might contribute to the superiority of the CVAER in RUL prediction as a combination of the two models.

Regarding the computation complexity of CVAER, the total trainable parameters and the average implementation time are summarized in Table 8. For a fully connected layer, the computation cost is an order of both input size *m* and output size *n*, O(mn). As for a 2D convolutional layer, the complexity is of order $O(ck^{2}hw)$, where *c* denotes the number of convolutional kernels, *k* is the kernel size, and *h*, *w* the dimensions of input height and width. To provide practical insights into computation costs, actual implementation time on the specified hardware is measured based on 15 cross-validation groups regarding training and testing phases. From Table 8, it is seen that the network requires a relatively longer time for training. Once training is finished, CVAER is capable of providing efficient and effective RUL predictions using a limited number of measurements, which exhibits potential for industrial applications by facilitating prompt maintenance planning once bearing faults are detected.

## 5. Discussion

Envelope analysis is a robust technique for bearing fault diagnosis and is widely acknowledged in industrial applications, whereas its potential to predict remaining useful life might have received less attention. In this work, the fault prognosis capability of envelope analysis is enabled by intelligent bearing RUL prediction. By modelling the latent uncertainty, the proposed CVAER is shown to be effective in providing probabilistic RUL prediction using multiple envelope spectra. Through the regularization mechanism between the encoder and the latent generator, the network effectively learns RUL-specific latent representations that enhance the accuracy and interpretability of RUL predictions. By leveraging both envelope analysis and uncertainty quantification, the reliability of the RUL prediction model is further improved, which is of practical significance in enhancing the applicability of DL-based RUL prediction to predictive maintenance.

To optimize the probabilistic framework, the evidence lower bound of the network is derived as the objective function, which is shown equivalent to the combination of the reconstruction loss of envelope spectra, the KL divergence regularizing the latent representation, and the regression loss for RUL prediction. Based on this formulation, the CVAER network not only provides a generative model for envelope spectra, but also learns regularized latent representations that improve the prediction performance. In addition, to enhance the generalization ability of the network, L2 regularization is introduced to balance the network complexity and the prediction accuracy by penalizing the large weights of the network. The regularization parameter is initially set to a small value and tuned based on the cross-validation results to ensure performance across implementations.

Regarding the application to different bearings, it is noted that the variability in material properties, manufacturing tolerances, and installation conditions may lead to different bearing degradation behaviours. To address the variability, the proposed method incorporates averaged envelope spectra to effectively capture the initiation and progression of defects, which provides a robust parameter set for RUL prediction. By utilizing life-cycle vibration data, the proposed method provides probabilistic RUL prediction with quantified uncertainty, offering insights for maintenance planning and decision-making. 

## 6. Conclusions

The bearing defect frequencies modulated around the bearing assembly resonance excited due to bearing defects are extracted using the envelope analysis. Hence, the envelope spectrum contains and reflects the bearing true conditions by removing dynamics related to the rotor. Hence, RUL estimation using the envelope spectrum once defects are detected is going to be reliable and useful for maintenance decisions. Thus, the proposed method is not simply based on raw data. The CVAER network is proposed for intelligent RUL prediction from multi-channel envelope spectra with quantified uncertainty to facilitate interpretable and trustworthy predictive maintenance of bearings. Thanks to its essence of generative modelling and probabilistic inference, the CVAER bridges the implicit mapping from averaged envelope spectra to bearing RUL distributions, which effectively extends the prognosis potential of envelope analysis beyond fault detection.

The benefits of the CVAER lie in the combination of the convolutional neural networks with variational auto-encoder for end-to-end RUL regression tasks, where the distribution of RUL is predicted for uncertainty quantification rather than a deterministic estimate, which provides a theoretical basis for assessing the confidence level of prediction to support practical decision-making. The two-dimensional convolutional layers are used in the CVAER where multi-channel envelope spectral input can be adopted efficiently. In addition, RUL-specific latent representations are effectively learned based on the regularization mechanism, which further elevates the interpretability of DL-based RUL prediction.

Through experimental and ablation studies, the proficiency of the CVAER in accurately predicting bearing RUL distributions after fault detection is demonstrated. For the generalization of the CVAER to a broader domain, future endeavours will focus on extending the applicability of the proposed method to variable operating conditions and exploring its adaptation for RUL prediction in diverse industrial systems.

## Figures and Tables

**Table 1:** Probabilistic properties of the CVAER network.

Network Components	Notation	Distribution	Parameterization
Probabilistic Encoder	ϕ	Q(z|X)	$N(z;f_{\phi }(X;\theta_{\phi }),{g_{\phi }(X;\theta_{\phi })}^{2}I)$
Decoder	ψ	$P\left(X|z\right)$	$N(X;f_{\psi }(z;\theta_{\psi }),{g_{\psi }(z;\theta_{\psi })}^{2}I)$
Probabilistic Regressor	Φ	Q(y|X)	$N(y;f_{\Phi }(X;\theta_{\Phi }),g_{\Phi }(X;\theta_{\Phi }))$
Latent Generator	Ψ	P(z|y)	$N(z;u^{T}y,\sigma^{2}I)$

**Table 2:** Test bearing specifications.

**Inner Raceway Diameter**	**Outer Raceway Diameter**	**Pitch Diameter**	**Contact Angle**
29.30 mm	39.80 mm	34.55 mm	0°
**Ball diameter**	**Number of balls**	**Basic dynamic load**	**Basic static load**
7.92 mm	8	12.82 kN	6.65 kN

**Table 3:** Accelerometer specifications.

**Model Number**	**Sensing Element**	**Sensitivity**	**Measurement Range**
PCB 352C33	Ceramic	100 mV/g	±50 g (peak)
**Resonant frequency**	**Frequency range (±5%)**	**Overload limit**	**Operating temperature**
≥50 kHz	0.5 to 10,000 Hz	±5000 g (peak)	−54 to +93 °C

**Table 4:** The measurement scheme and details of available run-to-failure datasets.

**Test Bearing Model**	**Sampling Rate**	**Sampling Length**	**Recording Interval**
LDK UER204	25,600 Hz	1.28 s (32,768 points)	Once per operating minute
**Condition: Speed/Loading**	**Bearing ID**	**Lifetime (minute)**	**Ending failure mode**
Condition 1: 35 Hz/12 kN	Bearing 1–1	123	Outer Race
	Bearing 1–2	161	Outer Race
	Bearing 1–3	158	Outer Race
	Bearing 1–4	122	Cage
	Bearing 1–5	52	Outer and Inner Races
Condition 2: 37.5 Hz/11 kN	Bearing 2–1	491	Inner Race
	Bearing 2–2	161	Outer Race
	Bearing 2–3	533	Cage
	Bearing 2–4	42	Outer Race
	Bearing 2–5	339	Outer Race
Condition 3: 40 Hz/10 kN	Bearing 3–1	2538	Outer Race
	Bearing 3–2	2496	Ball, Cage, Inner and Outer Races
	Bearing 3–3	371	Inner Race
	Bearing 3–4	1515	Inner Race
	Bearing 3–5	114	Outer Race

**Table 5:** FDT of the test bearings based on spectral inspection of the AES.

Condition: Speed/Loading	Bearing ID	FDT (min)	RUL at FDT (min)
C1: 35 Hz/12 kN	Bearing 1–1	66	57
	Bearing 1–2	35	126
	Bearing 1–3	59	99
	Bearing 1–4	45	77
	Bearing 1–5	29	23
C2: 37.5 Hz/11 kN	Bearing 2–1	446	45
	Bearing 2–2	47	114
	Bearing 2–3	128	405
	Bearing 2–4	8	34
	Bearing 2–5	121	218
C3: 40 Hz/10 kN	Bearing 3–1	2386	152
	Bearing 3–2	2334	162
	Bearing 3–3	327	44
	Bearing 3–4	1418	97
	Bearing 3–5	5	109

**Table 6:** Data and parameter settings for 15 cross-validation groups.

Group	Training and Validation	Testing	Group	Training and Validation	Testing
1	Bearing 1–2, 1–3, 1–4, 1–5, 1–5, 1–5	1–1	9	Bearing 2–1, 2–1, 2–1, 2–2, 2–2, 2–5	2–4
2	Bearing 1–1, 1–1, 1–3, 1–3, 1–3, 1–4	1–2	10	Bearing 2–1, 2–1, 2–2, 2–3, 2–3, 2–4	2–5
3	Bearing 1–1, 1–1, 1–2, 1–4, 1–5, 1–5	1–3	11	Bearing 3–2, 3–2, 3–3, 3–4, 3–4, 3–5	3–1
4	Bearing 1–1, 1–1, 1–2, 1–3, 1–5, 1–5	1–4	12	Bearing 3–1, 3–1, 3–3, 3–4, 3–5, 3–5	3–2
5	Bearing 1–1, 1–1, 1–1, 1–2, 1–3, 1–4	1–5	13	Bearing 3–1, 3–2, 3–4, 3–4, 3–4, 3–5	3–3
6	Bearing 2–2, 2–2, 2–3, 2–4, 2–4, 2–5	2–1	14	Bearing 3–1, 3–2, 3–3, 3–3, 3–3, 3–5	3–4
7	Bearing 2–1, 2–3, 2–4, 2–4, 2–4, 2–5	2–2	15	Bearing 3–1, 3–2, 3–2, 3–3, 3–4, 3–4	3–5
8	Bearing 2–1, 2–2, 2–4, 2–5, 2–5, 2–5	2–3			
	**Training: Validation**	**Learning rate**		**Mini-batch size**	**Iteration**
	70%:30%	10^−3^		64	500

**Table 7:** RUL prediction accuracy of different models based on one- or two-channel AES input.

RMSE (min)		Horizontal-Channel AES Input		2-Channel AES Input	
Group	Testing data	MLP	MLP-VAER [28]	CNN	CVAER (Proposed)
1	Bearing 1–1	23.47	15.05	14.46 *	**10.48**
2	Bearing 1–2	44.70	42.84 *	44.49	**41.57**
3	Bearing 1–3	27.21	24.30	22.11 *	**11.98**
4	Bearing 1–4	39.10	30.98 *	37.30	**28.71**
5	Bearing 1–5	47.07	41.94 *	44.03	**37.48**
6	Bearing 2–1	113.56	71.21	41.09 *	**30.75**
7	Bearing 2–2	72.49	34.92	33.32 *	**21.96**
8	Bearing 2–3	122.47	106.42	98.11 *	**66.65**
9	Bearing 2–4	55.90	49.28 *	52.79	**39.75**
10	Bearing 2–5	30.61	20.09 *	23.58	**11.81**
11	Bearing 3–1	42.33	41.67	41.14 *	**34.84**
12	Bearing 3–2	51.43	47.03 *	49.36	**46.50**
13	Bearing 3–3	45.92	26.98 *	34.89	**18.68**
14	Bearing 3–4	21.14	13.44 *	15.93	**11.08**
15	Bearing 3–5	31.29	22.35 *	27.96	**14.76**
Average RMSE		51.25	39.23	38.70 *	**28.47**

Precise RUL prediction has an RMSE of 0. The most accurate result is marked in bold font, and the runner-up models by ‘*’.

**Table 8:** Model complexity of CVAER and hardware implementation time.

Total Number of Trainable Parameters	Average Training Time (500 Iterations)	Average Testing Time (per Testing Set)	Average Testing Time (per Input Sample)
1,419,652	148.90 s	0.37 s	1.47 ms

## Data Availability

The bearing datasets are available at https://biaowang.tech/xjtu-sy-bearing-datasets/ accessed on 1 September 2022.

## Appendix A

**Algorithm A1.** A summary of CVAER algorithm.
**CVAER Algorithm**
** a. Training phase**
$\mathrm{Input}:\;\mathrm{Training}\;\mathrm{samples}:\;\mathrm{Multi}\text{-}\mathrm{channel}\;\mathrm{AES},\;X_{train}$$,\;\mathrm{RUL}\;\mathrm{label},\;{\hat{y}}_{train}$, learning rate, batch-size, iteration steps *N*
$\;\;\;\;\;\mathrm{Initialization}.\;\;\;\;\;\;\mathrm{For}\;\mathrm{iteration}\;\mathrm{number}\;i=1\;\mathrm{to}\;N:\;\;\;\;\;\;1.\;\mathrm{Compute}\;\mathrm{encoding}\;\mathrm{by}\;\mathrm{probabilistic}\;\mathrm{encoder}:\;u_{z},\;\log\Sigma_{z}=\phi (X_{train}^{\left(t\right)}).$ 2. Re-parameterize latent representation:z=μz+ϵΣz, ϵ~N0,I. $\;\;\;\;\;3.\;\mathrm{Compute}\;\mathrm{decoder}\;\mathrm{reconstruction}:\;X_{train}^{(t)\prime }=\psi (z).$ $\;\;\;\;\;4.\;\mathrm{Estimate}\;\mathrm{RUL}\;\mathrm{distributions}:\;\mu_{y},\;log\sigma_{y}^{2}=\Phi (X_{train}^{\left(t\right)}).$ $\;\;\;\;\;5.\;\mathrm{Generate}\;\mathrm{RUL}\text{-}\mathrm{specific}\;\mathrm{latent}\;\mathrm{representation}:\;u_{z}^{\prime },\;\log\Sigma_{z}^{\prime }=\Psi (y),\;\mathrm{where}\;y=\mu_{y}+\epsilon \sqrt{\sigma_{y}^{2}}.$ $\;\;\;\;\;6.\;\mathrm{Optimize}\;\mathrm{loss}:\;\min L=\min\left\{L_{Rec}\left(X_{train}^{\left(t\right)}\right)+L_{KL}\left(X_{train}^{\left(t\right)}\right)+L_{Label}\left(y||{\hat{y}}_{train}\right)\right\}\;\mathrm{based}\;\mathrm{on}\;\mathrm{Equations}\;(11)\text{–}(14).\;\;\;\;\;\;7.\;\mathrm{Backpropagation}\;\mathrm{and}\;\mathrm{update}\;\mathrm{model}\;\mathrm{parameters}\;\mathrm{via}\;\mathrm{Adam}\;\mathrm{optimizer}\;\mathrm{and}\;\mathrm{learning}\;\mathrm{rate},\;\alpha .$ $\;\;\;\;\;\mathrm{Until}\;\mathrm{meeting}\;\mathrm{termination}\;\mathrm{criteria}.\;\mathrm{Output}:\;\mathrm{Trained}\;\mathrm{CVAER},\;CVAER\left(\phi ,\psi ,\Phi ,\Psi \right).$
** b. Testing phase**
$\mathrm{Input}:\;\mathrm{Testing}\;\mathrm{samples}:\;\mathrm{Multi}\text{-}\mathrm{channel}\;\mathrm{AES},\;X_{test}$
$\;\;\;\;\;\mathrm{For}\;\mathrm{each}\;\mathrm{AES}\;\mathrm{sample}\;\mathrm{at}\;\mathrm{time}\;t,\;X_{test}^{\left(t\right)}\;\mathrm{in}\;\mathrm{Testing}\;\mathrm{set},\;X_{test}$ 1. Predict RUL distribution based on the shared encoder and probabilistic regressor, Φ: $\;\;\;\;\;\;\mathrm{RUL}\;\mathrm{distribution}:\;\mu_{y}$$,\;log\sigma_{y}^{2}=\Phi (X_{test}^{\left(t\right)}).$ $\;\;\;\;\;2.\;\mathrm{Generate}\;\mathrm{RUL}\text{-}\mathrm{specific}\;\mathrm{latent}\;\mathrm{representation}\;(\mathrm{optional}):\;u_{z}^{\prime }$$,\;\log\Sigma_{z}^{\prime }=\Psi (y),\;\mathrm{where}\;y=\mu_{y}+\epsilon \sqrt{\sigma_{y}^{2}}.$ $\mathrm{Output}:\;\mathrm{Predicted}\;\mathrm{bearing}\;\mathrm{RUL}\;\mathrm{with}\;\mathrm{quantified}\;\mathrm{uncertainty}\;\mathrm{from}\;\mu_{y}$$\;\mathrm{and}\;log\sigma_{y}^{2}$.
